# Intentions of Patients With Cancer and Their Relatives to Use a Live Chat on Familial Cancer Risk: Results From a Cross-Sectional Web-Based Survey

**DOI:** 10.2196/45198

**Published:** 2023-08-28

**Authors:** Paula Memenga, Eva Baumann, Hanna Luetke Lanfer, Doreen Reifegerste, Julia Geulen, Winja Weber, Andrea Hahne, Anne Müller, Susanne Weg-Remers

**Affiliations:** 1 Hanover Center for Health Communication Department of Journalism and Communication Research Hanover University of Music, Drama and Media Hannover Germany; 2 Bielefeld University Bielefeld Germany; 3 Krebsinformationsdienst Deutsches Krebsforschungszentrum Heidelberg Germany; 4 BRCA-Netzwerk Bonn Germany

**Keywords:** live chat, technology acceptance, familial cancer risk, Extended Unified Theory of Acceptance and Use of Technology, cancer information seeking, patients and relatives with cancer, patients with cancer, cancer risk, genetic testing, diagnosis, severity, cross-sectional survey

## Abstract

**Background:**

An important prerequisite for actively engaging in cancer prevention and early detection measures, which is particularly recommended in cases of familial cancer risk, is the acquisition of information. Although a lot of cancer information is available, not all social groups are equally well reached because information needs and communicative accessibility differ. Previous research has shown that a live chat service provided by health professionals could be an appropriate, low-threshold format to meet individual information needs on sensitive health topics such as familial cancer risk. An established German Cancer Information Service is currently developing such a live chat service. As it is only worthwhile if accepted by the target groups, formative evaluation is essential in the course of the chat service’s development and implementation.

**Objective:**

This study aimed to explore the acceptance of a live chat on familial cancer risk by patients with cancer and their relatives (research question [RQ] 1) and examine the explanatory power of factors associated with their intentions to use such a service (RQ2). Guided by the Extended Unified Theory of Acceptance and Use of Technology (UTAUT2), we examined the explanatory power of the following UTAUT2 factors: performance expectancy, effort expectancy, social influence, facilitating conditions, and habit, supplemented by perceived information insufficiency, perceived susceptibility, perceived severity, and cancer diagnosis as additional factors related to information seeking about familial cancer.

**Methods:**

We conducted a cross-sectional survey via a German web-based access panel in March 2022 that was stratified by age, gender, and education (N=1084). The participants are or have been diagnosed with cancer themselves (n=144) or have relatives who are or have been affected (n=990). All constructs were measured with established scales. To answer RQ1, descriptive data (mean values and distribution) were used. For RQ2, a blockwise multiple linear regression analysis was conducted.

**Results:**

Overall, 32.7% of participants were (rather) willing, 28.9% were undecided, and 38.4% were (rather) not willing to use a live chat on familial cancer risk in the future. A multiple linear regression analysis explained 47% of the variance. It revealed that performance expectancy, social influence, habit, perceived susceptibility, and perceived severity were positively associated with the intention to use a live chat on familial cancer risk. Effort expectancy, facilitating conditions, information insufficiency, and cancer diagnosis were not related to usage intentions.

**Conclusions:**

A live chat seems promising for providing information on familial cancer risk. When promoting the service, the personal benefits should be addressed in particular. UTAUT2 is an effective theoretical framework for explaining live chat usage intentions and does not need to be extended in the context of familial cancer risk.

## Introduction

### Live Chat for Providing Reliable, Tailored Cancer Information

Cancer is one of the leading causes of death worldwide [1], accounting for 1 in 5 deaths in Germany [2]. If cancer occurs more frequently within a family, this may be purely coincidental or can be attributed to a common lifestyle, similar environmental factors, or a hereditary predisposition, among other factors. Cancers that are attributable to lifestyle-related factors are preventable. Individuals with suspected hereditary cancer risk can have their risk determined by genetic testing and, on this basis, take risk-adapted prevention and early detection measures. If detected and treated early, there is a good chance of a cure for many tumors [3]. An important prerequisite for actively engaging in cancer prevention and early detection measures, which is particularly recommended in cases of a familial cancer risk, is the acquisition of information [4,5]. Accordingly, cancer information-seeking behavior—that is, actively seeking, evaluating, and interpreting cancer information [6]—of patients with cancer and their relatives is crucial for their health and the health of their families, respectively [7].

Although there is already a lot of cancer information available, not all social groups are equally well reached because information needs and access differ, for example, due to a lack of barrier-free information or the users’ insufficient health literacy [8,9]. In particular, there is a need for reliable, tailored cancer information [10] that addresses individual risks, risk-adapted prevention, early detection measures, and treatment options. Previous research has shown that a live chat service could be an appropriate format to meet individual information needs on sensitive health topics such as cancer [11-13] and reach distinctly vulnerable groups, for example, people with a migration background [14]. Using a live chat, inquirers benefit from personalized information and easy access to reliable health and medical expertise, irrespective of where they are located [15]. Due to the high level of visual and auditory anonymity, users may experience an increased sense of security as they can hide their emotions [11]. Moreover, writing instead of talking provides more time to think and compose thoughts and further questions [11].

Against this background, the German Cancer Information Service (Krebsinformationsdienst) of the German Cancer Research Center (Deutsches Krebsforschungszentrum) is currently expanding its information services to implement a web-based real time written chat for providing tailored information on familial cancer risk. This live chat enables synchronous, 1-to-1 written conversations between physicians of the Cancer Information Service and individuals interested in cancer information (eg, patients with cancer and their relatives) in a web-based environment (ie, on a website or via a mobile phone app). It focuses on individual information about genetic and lifestyle-related cancer risks, as well as, the promotion of appropriate coping strategies, especially in the form of prevention and early detection measures. The chat is therefore aimed at interested individuals in Germany who would like general information on familial cancer risk, prevention, or early detection, but especially at individuals who suspect an increased risk of cancer due to an accumulation of individuals with cancer in the family or who already know about a genetic predisposition.

However, such a live chat requires many resources on the part of the provider, needs to be adjusted to the users’ preferences, and is only worthwhile if accepted by the target groups. Formative evaluation is thus essential in the course of the chat’s development and implementation. Guided by the Extended Unified Theory of Acceptance and Use of Technology (UTAUT2) [16], this study aimed to explore the acceptance of a live chat on familial cancer risk by patients with cancer and their relatives (research question [RQ] 1) and identify factors associated with their intentions to use such a service (RQ2), using a formative approach. In particular, we examined the explanatory power of the following UTAUT2 factors: performance expectancy, effort expectancy, social influence, facilitating conditions, and habit, and the additional explanatory power of perceived information insufficiency, perceived susceptibility, severity of familial cancer risk, and a cancer diagnosis by oneself as additional factors related to information seeking about familial cancer.

### Modeling Factors Associated With the Intention to Use a Live Chat on Familial Cancer Risk

#### UTAUT2 Factors

A widely accepted theory for explaining consumers’ intentions to use digital technologies such as live chats is the UTAUT2 [16], which has already proven effective in diverse health contexts such as teleconsultation technology [17,18], electronic patient portals [19-21], or mobile health apps [22-26]. It is an extension of the Unified Theory of Acceptance and Use of Technology [27], which was originally developed in an organizational context and integrates elements of 8 theoretically sound models from the fields of psychology, for example, the Theory of Planned Behavior, sociology, for example, the Innovation Diffusion Theory, and information systems research, for example, the Technology Acceptance Model. The UTAUT2 postulates performance expectancy, effort expectancy, social influence, facilitating conditions, hedonic motivation, price value, and habit as predictors of usage intentions.

In the health context, performance expectancy is related to the expected personal benefit of using a specific technology such as a live chat on familial cancer (eg, meeting information needs and supporting decision-making). It is one of the strongest predictors of behavioral intentions having a moderate to large effect [17,18,20,22,23,25]. Effort expectancy refers to the expected ease of use of digital technology. It thus describes, for example, the extent to which a person perceives a live chat interaction as clear and understandable [21,25,28]. Social influence covers subjective norms, that is, the individual’s perception that important others, whose opinion the respondent values, expect him or her to behave in a certain way [29]. It is also a key predictor in health information–seeking behavior models (eg, [30]), known to be a driver of health-related behaviors such as seeking information [31] or using health-related technologies [20,21,26,32]. Facilitating conditions consider the available resources and external support for using a specific technology. In the context of a live chat, a facilitating condition would be, for example, the fulfillment of technical requirements to use it [20,23,28]. Habit refers to the extent to which the use of technology occurs automatically based on prior experiences [16]. It has rather small effects in the context of less common technologies such as telemedicine interventions [17,18], and moderate to strong effects in terms of mobile apps, which are likely to be used more frequently in everyday life outside of health care [23,24,26].

As the use of health-related technology is often free of charge and medical contexts require a sensitive approach that is not necessarily enjoyable, the UTAUT2 factors hedonic motivation and price value have often not been considered in contexts of health [18,20,23,33] and were also excluded in this study.

In line with the current state of research, we postulated the following hypothesis:

H1: Performance expectancy (H1a), effort expectancy (H1b), social influence (H1c), facilitating conditions (H1d), and habit (H1e) are positively associated with the intention to use a live chat on familial cancer risk.

#### Factors Related to Information Seeking About Familial Cancer

Since the UTAUT2 contains mostly application-centered factors and is often criticized for not being specific enough to the health context [34], we aimed to extend the model by integrating factors that are derived from established health information–seeking behavior models and could additionally drive live chat usage intentions.

First, information insufficiency, which is the perceived information need regarding familial cancer risk, was integrated as an additional factor. It describes the difference between the perceived current level of knowledge and the perceived required level to adequately deal with familial cancer risk; it is a frequently considered predictor of health- and risk-related information seeking [30,35,36]. The assumption that perceiving a gap between the 2 levels of knowledge drives information seeking is based on the motivation for accuracy [35,37], that is, the willingness to gain a sufficient understanding of information and form accurate judgments. Previous studies have shown both positive associations between information insufficiency and health information seeking [38,39] as well as no associations between the 2 constructs [30,39-41]. Therefore, Link et al [39] suggested a contextual role of perceived knowledge insufficiencies, proposing that they influence health information seeking when facing health threats of high personal relevance. Thus, in the context of familial cancer risk, which is associated with high levels of fear and uncertainty [42], information insufficiency may be positively associated with the intention to use a live chat to meet information needs and receive decision-making support.

Second, risk perceptions about familial cancer risk were integrated. Risk perceptions describe an individual’s perceived susceptibility and severity, that is, the likelihood of being affected by a familial cancer risk and its threat assessment that increases the salience of the disease [30]. They are central antecedents of the intention to use digital services [22,33,43] for cancer-related information seeking [38,44] and the adoption of healthy behaviors in general [45]. Accordingly, individuals who perceive higher susceptibility and severity related to familial cancer risk may be more likely to have the intention to use live chat on familial cancer risk.

Finally, a cancer diagnosis could be an additional factor associated with the intention to use the live chat. Lower health status is related to more frequent health-related information seeking in general [39,46,47] and is thus considered in various models of health technology acceptance [43,48]. With regard to the cancer context, former research suggests that being personally affected by cancer is a driver of cancer-related information seeking [49], which leads to the following hypothesis:

H2: Beyond the UTAUT2 factors, information insufficiency (H2a), perceived susceptibility (H2b), perceived severity (H2c), and a cancer diagnosis (H2d) provide additional explanatory power for the intention to use a live chat on familial cancer risk (positive effects assumed).

**Figure 1:**
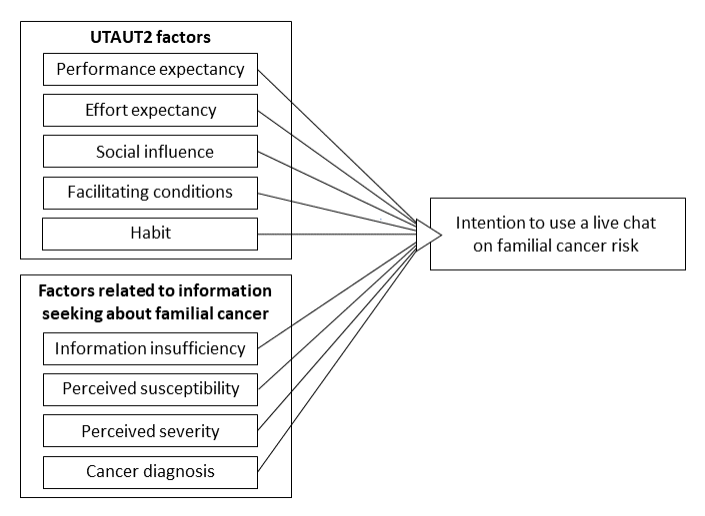
Research model. Age, gender, and education level were included as control variables. UTAUT2: Extended Unified Theory of Acceptance and Use of Technology [16].

An overview of the proposed research model is shown in Figure 1.

## Methods

### Participants, Recruitment, and Sampling

To test our hypotheses and answer our RQ, we conducted a cross-sectional web-based survey with a sample of 1100 German individuals from March 16, 2022, to March 22, 2022. All participants were internet users aged 18 years and older, and are currently or have been in the past diagnosed with cancer themselves or have relatives (life partner or blood relatives including children, siblings, parents, grandparents, aunts or uncles, nieces or nephews, cousins) who are or have been affected by this disease and thus could be potentially interested in using the live chat. The fieldwork was conducted by the German market research institute Cint/GapFish using quota sampling. The sample was stratified by gender (50% men, 50% women), age (population-representative distribution), and education (50% lower level of education including general certificate of upper secondary education, 50% higher level of education, that is, at least Abitur). Participants were invited until the desired sample size and quotas were reached. To determine whether they fit the target group and quotas, they were first asked about their personal and familial cancer history and provided information about their gender, age, and level of education. Individuals who did not fit the target group and quotas were excluded from further questioning. The study is described according to the CHERRIES (Checklist for Reporting Results of Internet E-Surveys) guidelines [50].

### Sample Size and Power

The intended sample size was 1100. A statistical a priori power analysis with G*Power (Kiel University) showed that this sample size is sufficient for a multiple linear regression analysis with 12 factors to reveal small to moderate effects with a statistical power of 0.95 (*f²*=0.085, α=.05). During data cleaning, 16 individuals who gave implausible answers or indicated that they did not understand some of the questions were excluded. Thus, the final sample size was 1084.

### Measures

#### Intention to Use

To measure the intention to use a live chat on familial cancer risk, participants were first given an introductory text to the planned live chat, which included information about the topic and target group of the chat, as well as about how it would work. The introduction also included an image of the live chat integrated into a website with further information (see Multimedia Appendix 1). Subsequently, participants were asked about their intentions to use such a service. Usage intentions were measured with 3 items adapted from the original UTAUT2 scale [16] (see Multimedia Appendix 2). The participants assessed their responses on a 5-point Likert-type scale, ranging from 1=strongly disagree to 5=strongly agree. The scale was summarized into a mean index (α=.94; mean 2.89, SD 1.13).

#### UTAUT2 Factors

Performance expectancy, effort expectancy, social influence, facilitating conditions, and habit were measured using a modified version of the original UTAUT2 scale [16] (see Multimedia Appendix 2). Performance expectancy, social influence, and habit were each measured with 3 items, and effort expectancy and facilitating conditions were each measured with 4 items on a 5-point Likert-type scale (1=strongly disagree to 5=strongly agree). Mean indices were calculated for all scales (performance expectancy α=.88, mean 3.46, SD 0.97; effort expectancy α=.94, mean 4.17, SD 0.90; social influence α=.93, mean 2.98, SD 1.04; facilitating conditions α=.78, mean 3.93, SD 0.85; habit α=.91, mean 3.26, SD 1.22).

#### Factors Related to Information Seeking About Familial Cancer

To measure the participants’ information insufficiency, they were first given a brief explanation of familial cancer risk:

An increased familial cancer risk may be present if, for example, several members of a family develop or have developed cancer, or if a person develops the disease at a young age. In addition to a common lifestyle or similar environmental factors, one reason for an accumulation of cancer cases in the family can also be a hereditary predisposition.

Following Kahlor [30], they subsequently rated their perceived current knowledge and desired knowledge on different scales of 0 (no knowledge at all or need to know nothing) to 100 (comprehensive knowledge or need to know everything). For information insufficiency, perceived current knowledge was subtracted from the desired knowledge. Higher values of the created variable mean higher information insufficiency (mean –17.31, SD 26.45; minimum=–100; maximum=100).

The perceived susceptibility (mean 3.55, SD 1.27) and severity (mean 3.15, SD 0.91) of familial cancer risk were measured with single items adapted from Kahlor [30] (see Multimedia Appendix 2). Both items were assessed on 5-point Likert-type scales, ranging from 1=not at all likely or threatening to 5=extremely likely or threatening. To measure a cancer diagnosis, the participants were asked if they are or have been affected by cancer (yes or no).

### Data Analysis

To answer the first RQ, descriptive data (mean values and distribution) were used. For the second RQ, a blockwise multiple linear regression analysis was conducted to examine the explanatory power of the UTAUT2 factors (H1) and factors related to information seeking about familial cancer (H2) for the intention to use the live chat. In the first block, sociodemographic factors were included as control variables. In the second block, the UTAUT2 factors were considered, whereas, in the third block, additional factors related to information seeking about familial cancer were integrated. All variables were included in the model based on theoretical assumptions.

### Ethics Approval

The study received ethical approval from the Central Ethics Committee at Leibniz University Hannover, Germany (EVLUH20/2021). All participants were informed of the investigator, purpose, content, data storage, and duration of the survey on the first page of the questionnaire and provided informed consent before participating. All study data are anonymous. The participants were financially compensated by the market research institute that conducted the survey.

## Results

### Participant Characteristics

The 1084 participants who were included for data analysis are currently or have been in the past diagnosed with cancer themselves (n=144) or have relatives who are or have been affected by this disease (n=990). They were aged between 18 and 87 (mean 47.60, SD 15.92) years. Overall, 547 (50.5%) were female and 537 (49.5%) were male. In addition, 543 (50.1%) had a low level of education and 541 (49.9%) were highly educated.

### Acceptance of the Live Chat

The first RQ addressed the acceptance of a live chat on familial cancer risk by patients with cancer and their relatives. Overall, the participants showed a moderate intention to use the live chat (mean 2.89, SD 1.13). A total of 355 (32.7%) participants were (rather) willing, 313 (28.9%) were undecided, and 416 (38.4%) were (rather) not willing to use such a service in the future.

Additional exploratory analyses for sociodemographic characterization of the 3 subgroups revealed that the average age in the uninterested subgroup was slightly higher (mean 51.45, SD 16.63 years) than in the undecided (mean 46.36, SD 15.34 years) and interested (mean 44.16, SD 14.62 years) groups (*F*_2,1081_=22.21; P<.001; partial η²=0.04). Moreover, the proportion of people with high education level was higher in the group of interested people (44.2% low-educated vs 55.8% high-educated) and lower in the groups of uninterested (53.6% low-educated vs 46.4% high-educated) and undecided (52.1% low-educated vs 47.9% high-educated) people (*χ*²_2_=7.4; P=.02; φ=0.08). In contrast, the subgroups did not differ with respect to gender (uninterested 51.9% female, undecided 51.1% female, interested 48.3% female; *χ*²_2_=1.1; P=.59; φ=0.03).

### Factors Associated With the Intention to Use the Live Chat

The second RQ focused on factors associated with the intention to use a live chat on familial cancer risk and their explanatory power. The overall model of the blockwise multiple linear regression analysis was significant (*F*_12,1070_=80.88, P<.001) and explained 47% of the variance in the intention to use the live chat. The results are presented in Table 1.

**Table 1:** Factors associated with the intention to use a live chat on familial cancer risk (N=1083).^a^

Factors			*B*	95% CI for *B*				SE *B*		β		*R*²_*corr*_			Δ*R*²_*corr*_	
				LL^b^		UL^c^										
Constant			–0.93	–1.39		–0.47		0.23		N/A^d^		N/A			N/A	
**Sociodemographic factors**													0.044			0.044^e^
	Age	0		–0.004	0.003		0		0		N/A			N/A		
	Gender (reference: female)	0.10		0.003	0.20		0.05		.05^f^		N/A			N/A		
	Education level (reference: low)	0.07		–0.03	0.17		0.05		.03		N/A			N/A		
**UTAUT2^g^ factors**													0.436			0.392^e^
	Performance expectancy	0.29		0.21	0.36		0.04		.25^e^		N/A			N/A		
	Effort expectancy	–0.01		–0.10	0.09		0.05		–.01		N/A			N/A		
	Social influence	0.29		0.23	0.36		0.03		.27^e^		N/A			N/A		
	Facilitating conditions	0.03		–0.07	0.13		0.05		.03		N/A			N/A		
	Habit	0.21		0.16	0.26		0.03		.23^e^		N/A			N/A		
**Factors related to information seeking about familial cancer**													0.470			0.034^e^
	Information insufficiency	0		–0.002	.002		0		0		N/A			N/A		
	Perceived susceptibility	0.07		0.03	0.11		0.02		.08^e^		N/A			N/A		
	Perceived severity	0.22		0.16	0.28		0.03		.18^e^		N/A			N/A		
	Cancer diagnosis (reference: yes)	–0.07		–0.22	0.09		0.08		–.02		N/A			N/A		

^a^The table shows model 3 of the blockwise multiple linear regression analysis. The overall model was significant (*F*_12,1070_=80.88, P<.001) and explained 47% of the variance. All variance inflation factors (VIF)<5.

^b^LL: lower limit.

^c^UL: upper limit.

^d^N/A: not applicable.

^e^*P*≤.001 (exact P values can be found in the text).

^f^P<.05 (exact P values can be found in the text).

^g^UTAUT2: Extended Unified Theory of Acceptance and Use of Technology.

The first block which included age, gender, and education level as control variables contributed to 4.4% of the explained variance. Gender was positively, albeit weekly, associated with the intention to use the live chat (β=.05, P=.043). Thus, men tend to be more willing to use the chat than women. Age (β=0, P=.90) and education level (β=.03, P=.18) were not related to usage intentions.

The second block included the UTAUT2 factors. In H1, we assumed that performance expectancy (H1a), effort expectancy (H1b), social influence (H1c), facilitating conditions (H1d), and habit (H1e) are positively associated with the intention to use the live chat. Indeed, these factors accounted for the greatest share of explained variance (Δ*R*^2^=0.392). Performance expectancy (β=.25, P<.001), social influence (β=.27, P<.001), and habit (β=.23, P<.001) were positively associated with the intention to use the live chat. Thus, individuals who expect a higher personal benefit from using the live chat, individuals who perceive that important others expect them to use the service, and individuals who use live chats more frequently in their daily lives, tended to have higher intentions to use the live chat on familial cancer risk. In contrast, effort expectancy (β=–.01, P=.84) and facilitating conditions (β=.03, P=.51) were not significantly associated with the intention to use the live chat. Thus, hypotheses H1a, H1c, and H1e were accepted, whereas hypotheses H1b and H1d had to be rejected.

The third block focused on factors related to information seeking about familial cancer. H2 postulated that information insufficiency (H2a), perceived susceptibility (H2b), perceived severity (H2c), and a cancer diagnosis (H2d) are additionally positively associated with the intention to use the live chat on familial cancer risk. The inclusion of these variables increased the amount of explained variance by only 3.4%. Perceived susceptibility (β=.08, P<.001) and perceived severity (β=.18, P<.001) were positively associated with the intention to use the live chat. Accordingly, individuals who perceive a higher likelihood of having a familial cancer risk and who perceive such a risk to be more threatening tend to have higher intentions to use the live chat on familial cancer risk. Information insufficiency (β=.00, P=.96) and a cancer diagnosis (β=–.02, P=.39), on the other hand, did not contribute to the explanation of the intention to use the live chat. Thus, hypotheses H2b and H2c were accepted, whereas hypotheses H2a and H2d had to be rejected.

## Discussion

### Principal Findings on Live Chat Acceptance

This study explored the intentions of patients with cancer and their relatives to use a live chat on familial cancer risk and examined the explanatory power of potential associated factors. With regard to the first RQ on live chat acceptance, the survey results suggest that about one-third of the participants are interested in using such a service in the future. Since the live chat is limited in terms of subject matter and individuals have different channel-specific preferences for obtaining health- and cancer-related information, this finding can certainly be considered promising. It is also in line with the findings of a chat evaluation in the context of cancer genetic testing, which revealed an actual usage rate of 32% [12].

The high number of undecided participants indicates that it may have been difficult for some to decide whether or not they would use the live chat based only on a description and without trying it out. In addition, the variance in usage intentions demonstrates that individual cancer information and communication preferences may vary, underlining the need for different communication strategies in order to reach a broad target group. Another explanation could be that the variance hides different subgroups that differ in their intentions to use the chat. Our results provide initial evidence of sociodemographic differences with respect to age and education level. However, these differences lost relevance when the other factors were integrated into the model.

### Key Findings and Theoretical Implications

#### Role of the UTAUT2 Factors

Regarding the second RQ, our study identified performance expectancy, that is, the perceived usage and expected benefits, social influence of important others whose opinions the participants value, and habit of using live chats in everyday life as the strongest associated and thus determining factors of the intention to use the live chat service. This is in line with previous research, that has often identified performance expectancy [22,23,25,51] and habit [23,24,51] as the strongest predictors of health-related technology usage intentions. In addition, social influence seems particularly crucial in the familial cancer context. Because familial cancer risk has high relevance for the whole family and is generally associated with high levels of uncertainty and anxiety, this topic is likely to be discussed a lot with family members, who are among the most preferred sources of cancer-related advice [52].

However, the perceived ease of use, as manifested by effort expectancy and facilitating conditions, did not seem to be relevant in the context of using a cancer-related live chat. In line with our findings, recent studies on usage intentions of health-related technology revealed that fulfillment of technical requirements has a rather subordinate significance [17,20,22,24,26]. Thus, having the necessary resources and technical support might only become decisive in the actual usage situation of the live chat [27]. Further, this result could also be related to the fact that we used a web-based access panel whose members presumably have few difficulties overall with digital services such as chat [15].

#### Role of Information Insufficiency, Risk Perceptions, and Cancer Diagnosis

Beyond the UTAUT2 factors, several additional theoretically based and empirically supported associated factors contributed to the explanation of live chat usage intentions. In line with our hypothesis, perceived susceptibility and severity of a (potential) familial cancer risk were positively associated with the intention to use the live chat. In particular, participants who perceived a higher threat showed higher usage intentions, whereas the perceived likelihood of being affected by a familial cancer risk seemed to have a subordinate importance. Perceived susceptibility, which may be associated with fear [30], is a stronger driver of usage intentions than factual probability assessment. Thus, the chat is likely to be used by people who are concerned about a family history of cancer.

Other factors proved not to be relevant. Information insufficiency, that is, the perceived lack of knowledge regarding familial cancer risk, was not associated with the intention to use the live chat. Considering our theoretical derivation of information insufficiency as a frequently considered predictor in health information seeking models [30,36], this is surprising. Further, given that previous studies have found both positive [38] and no effect [41] of information insufficiency on cancer risk information seeking, our result cannot be explained by context as suggested by Link et al [39]. However, our dependent variable—intention to use a live chat—provides a possible explanation. Feeling inadequately informed could generally drive cancer information seeking but does not necessarily explain the selection of a particular information channel.

As opposed to our hypothesis, a cancer diagnosis was not associated with higher intentions to use the live chat. Individuals with a cancer diagnosis and those who are not themselves affected by cancer but have at least 1 case of cancer in their family did not differ in their intentions to use the live chat, indicating similar personal importance of familial cancer risk in both groups. This demonstrates that not only affected individuals themselves but also relatives of patients with cancer require information on familial cancer risk and their needs should be addressed with communication and information measures.

In summary, the UTAUT2 is an effective theoretical framework for explaining the intention to use a live chat service on familial cancer risk. Other contextual factors related to information seeking about familial cancer were of minor importance and had only little explanatory power. From our results, there is no need to expand the UTAUT2 in the context of a live chat on familial cancer risk.

### Limitations and Direction for Future Research

This study is not without limitations and provides a starting point for further research. First, the survey was a cross-sectional survey and does not allow for causal inferences. Future studies should use longitudinal and experimental designs to examine the reciprocal effects of perceptions, expectations, and usage intentions. Second, our sample consisted of overall more digitally savvy people limiting the generalizability of our results. This should be considered when interpreting the results, as less digitally savvy people are likely to have more difficulties using health-related technologies, which could decrease their intentions to use such a service [23]. Thus, the acceptance of less digitally savvy people needs to be researched in future studies. Third, the type of cancer was not considered in this study. However, in the case of certain cancers in which hereditary factors play a central role, the issue of familial cancer risk may be more salient, which could influence usage intentions; thus, it should be considered in future studies. Fourth, participants were asked to assess their live chat usage intentions that may differ from the actual use and experiences. Future studies should examine the entire process from the intention to use, to the actual usage and user experience, followed by the analysis of the impacts and benefits of using a live chat on familial cancer risk. In addition, more differentiated analyses are required on the specific information needs, expectations, and requirements of different groups of society, such as persons with varying levels of education or migrant background to address different target groups and provide tailored information support via the live chat.

### Conclusion and Practical Implications

In conclusion, a live chat appears promising for providing information on familial cancer risk. However, as individual information and communication preferences vary, it is reasonable to combine different information channels and communication strategies to reach a broader target group and achieve the greatest effects in cancer prevention and early detection. When promoting live chat, the personal benefits of using such a service should be addressed. In addition, the social environment seems to have an important influence suggesting that not only the advice seekers themselves but their family members and friends should also be addressed regarding the benefits of such a live chat service. Since the habit of using live chats is also of central importance for usage intentions, comparisons with chat formats familiar in everyday life such as instant messaging services could be made to lower the inhibition threshold of use. Moreover, people who perceive a high threat of a (potential) familial cancer risk and contact other services (eg, primary care physician and health insurance provider) should be made aware of the availability of live chat services on familial cancer risk by the respective health care providers. Finally, health care providers should increase education about the risk of a cluster of cancers in a family to emphasize the importance of appropriate prevention and early detection measures and to highlight the relevance of the live chat on familial cancer risk—even for individuals who have not previously perceived a familial risk of cancer.

## Appendix

Multimedia Appendix 1 Introduction live chat.

Multimedia Appendix 2 Survey items.

## Abbreviations

CHERRIES: Checklist for Reporting Results of Internet E-Surveys
RQ: research question
UTAUT2: extended unified theory of acceptance and use of technology

## Data Availability

Data are available upon reasonable request from the corresponding author.
