# Supplementary material for: Intentions of Patients With Cancer and Their Relatives to Use a Live Chat on Familial Cancer Risk: Results From a Cross-Sectional Web-Based Survey
Source: J Med Internet Res. 2023 Aug 28;25:e45198. doi: 10.2196/45198 (PMC10495847; doi:10.2196/45198)

**Appendix 1**

**Introduction of the Live Chat**

The Cancer Information Service would like to offer another information service within the near future. It will be a chat specifically on familial cancer risk. In the following, we will give you a short overview of the chat. Please read the following text carefully.

**How does the chat work?**

Using the chat, you will have the opportunity to make individual contact with a physician or experienced patient representative online through a chat window on a website. You can exchange written messages in real-time and ask all your individual questions on family cancer risk. The messages cannot be read by third parties, but only by you and the physician/patient representative.

**Who is the chat for?**

The chat is for cancer patients, their relatives, as well as interested individuals who have questions on familial cancer risk in general. Unfortunately, questions on other cancer-related topics cannot be answered in the chat (but they will be forwarded to the telephone or e-mail service).

**What does the chat look like?**

The following image shows how such a chat on the website will look like.


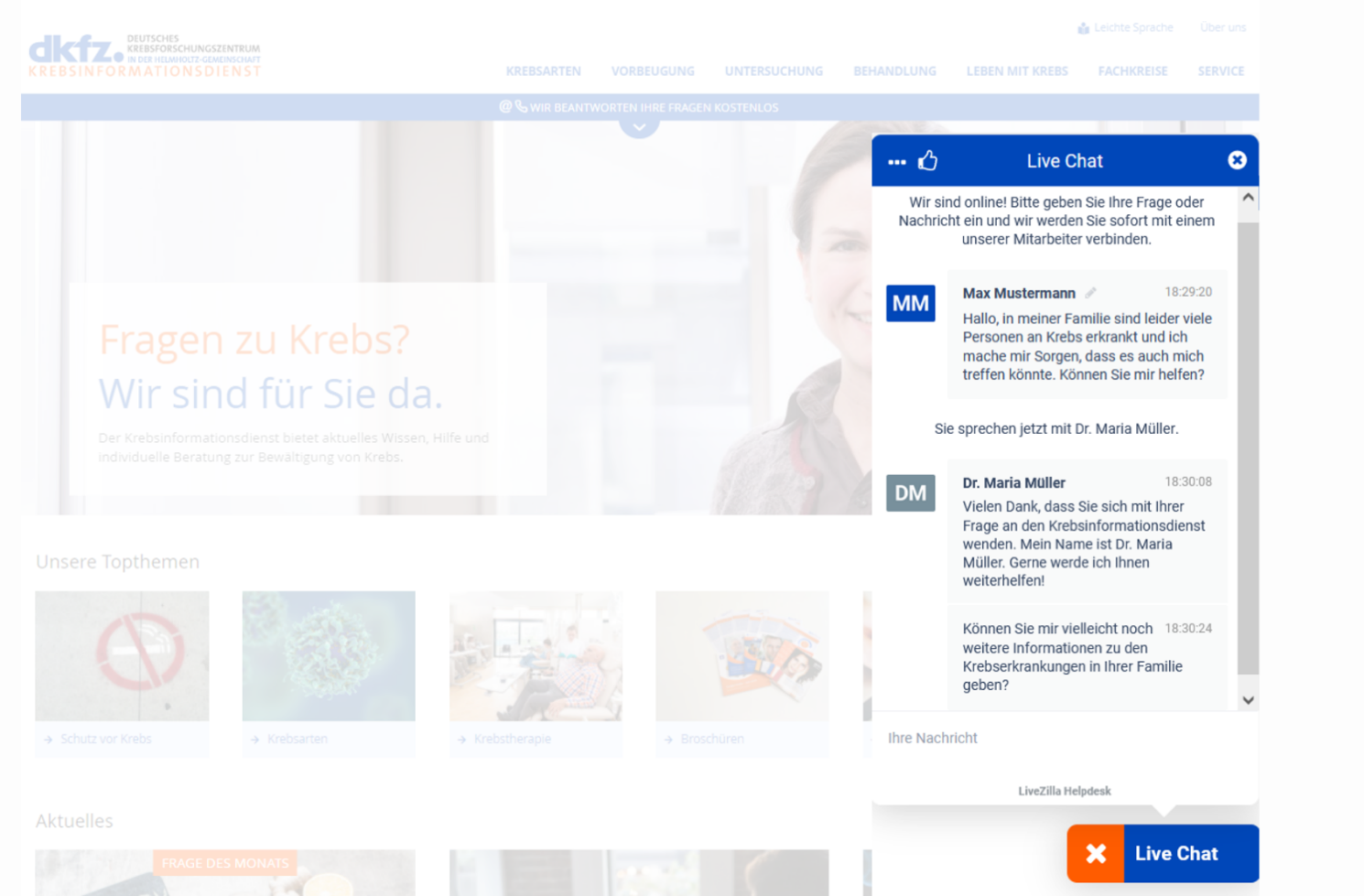

Supplement: Multimedia Appendix 1 [file jmir_v25i1e45198_app1.docx]
