# Supplementary material for: Intentions of Patients With Cancer and Their Relatives to Use a Live Chat on Familial Cancer Risk: Results From a Cross-Sectional Web-Based Survey
Source: J Med Internet Res. 2023 Aug 28;25:e45198. doi: 10.2196/45198 (PMC10495847; doi:10.2196/45198)
Supplement: Multimedia Appendix 2 [file jmir_v25i1e45198_app2.docx]

**Appendix 2**

**Survey Items**

| **Construct** | **Items** | **Source** |
| --- | --- | --- |
| Intention to use a live chat | *BI1.* I intend to use the chat in the future.  *BI2.* I think I would use the chat.  *BI3.* I am interested in using the chat. | Venkatesh et al., 2012 |
| Performance expectancy | *PE1.* I expect the chat to be of useful to me.  *PE2.* Using the chat will help me to make decisions related to my health.  *PE3.* Using the chat will help me to meet my information needs about family cancer risk. |  |
| Effort expectancy | *EE1.* Learning how to use the chat is easy for me.  *EE2.* My interaction with the chat is clear and understandable.  *EE3.* I expect the chat will be easy to use.  *EE4.* I expect to deal well with the chat. |  |
| Social influence | *SI1.* People who are important to me think that I should use the chat.  *SI2.* People who influence my behavior think that I should use the chat.  *SI3.* People whose opinions that I value prefer that I use the chat. |  |
| Facilitating conditions | *FC1.* I have the resources necessary to use the chat (e.g., computer/smartphone, Internet).  *FC2.* I have the knowledge necessary to use the chat.  *FC3.* The chat is compatible with other technologies I use.  *FC4.* I can get help from others when I have difficulties using the chat. |  |
| Habit | *HT1.* The use of chats has become a habit for me.  *HT2.* I use chats frequently in my everyday life.  *HT3.* I generally like using chats. |  |
| Current level of knowledge | Rate your knowledge about familial cancer risk on a scale of 0 to 100, where zero means knowing nothing about it and 100 means knowing everything you could possibly know about familial cancer risk. | Kahlor, 2010 |
| Desired level of knowledge | Think of that same 0 to 100 scale again. This time, estimate how much knowledge you need to be adequately informed about familial cancer risk. |  |
| Perceived susceptibility | How likely do you think it is that there is a familial cancer risk in your family? |  |
| Perceived severity | If there is or would be a familial cancer risk in your family, how serious is this to you? |  |
| Cancer diagnosis | Have you received a cancer diagnosis currently or in the past? | --- |
